# Dependence of Tape-Worm on the Use of Raw or Underdone Meat

**Published:** 1859-08

**Authors:** 


*Dependence of Tape-Worm on the use of Raw or Underdone Meat.*—The interesting discoveries of Von Siebold and of Küchenmeister, and their speculations as to the various modes in which the ova of intestinal worms enter the human body, induced Dr. J. Barclay, Physician to the Leicester Infirmary, to inquire of all his tape-worm patients, during the last twelve wonths, whether they were fond of meat so underdone as to be a possible vehicle of the germs. In every one of the ten cases observed by him, he ascertained that not only underdone, but really absolutely raw meat had been eaten. In some instances the fact was only elicited by cross-questioning; in others it was at once acknowledged.—*Med. Times and Gaz*, March 26, 1859.

